# Trends in Race and Ethnicity Among Matriculants to US Oncology Training Programs, 2015-2020

**DOI:** 10.1001/jamanetworkopen.2021.28530

**Published:** 2021-10-07

**Authors:** Jacob J. Lang, Rochell Issa, Conner Lombardi, Emilie Garcia, Oluchi C. Oke, Obi Ekwenna

**Affiliations:** 1 University of Toledo College of Medicine and Life Sciences, Toledo, Ohio; 2 Department of General Oncology, University of Texas MD Anderson Cancer Center, Houston; 3 Office of Student Affairs & Department of Urology and Transplantation, University of Toledo College of Medicine and Life Sciences, Toledo, Ohio

## Abstract

This cross-sectional study assesses underrepresented minority trends across oncology specialties from 2015 to 2020.

## Introduction

The demographics of practicing physicians in the US have not represented the diverse populations they serve, and there is no exception in oncology care.^1,2^ Recognizing the detrimental effects of these disparities on public health, oncology training programs have instilled benchmarks and initiatives to increase diversity among oncology physicians nationwide.^3^ However, data about representation in oncology specialties and the impact of these initiatives is lacking. This study aims to assess underrepresented minority (URM; ie, Black or African American individuals, Hispanic or Latino individuals, and Native American, American Indian, or Alaskan Native individuals) representation trends across training programs in oncology specialties from 2015 to 2020.

## Methods

This cross-sectional study was exempt from institutional review board review at the University of Toledo and informed consent was waived because data was deidentified. This study follows the Strengthening the Reporting of Observational Studies in Epidemiology (STROBE) reporting guideline.

This study uses trainee self-reported race and ethnicity data collected by the Accreditation Council for Graduate Medical Education (ACGME) from the 2015 to 2016 to the 2019 to 2020 academic calendar years.^4^ Trainees self-identified as Asian or Pacific Islander (or Native Hawaiian in 2019-2020); Black or African American; Hispanic or Latino; Native American, American Indian, or Alaskan Native; White; or other. Per ACGME resource books, the category other indicates a known ethnicity other than the ones available in the selection. Trainees were grouped into all oncology specialties, including hematology and medical oncology, gynecologic oncology, pediatric hematology and oncology, radiation oncology, complex general surgical oncology, and all other specialties. χ^2^ analysis was used to compare URM representation, which was defined as the proportion of trainees self-identifying as Black or African American, Hispanic or Latino, or Native American or Alaskan, across groups. Joinpoint regression was used to determine Average Annual Percentage Change (AAPC) in representation for all groups.^5^ Joinpoint Regression Software was used to calculate AAPC. Stata Statistical Software version 14.2 (StataCorp) was used for χ^2^ analysis. All tests were 2-tailed. Significance was determined at *P* < .05.

## Results

Of the 13 853 total oncology trainees included in this study, 4315 trainees (31.1%) identified as Asian or Pacific Islander (or Native Hawaiian in 2019-2020); 520 trainees (3.8%) identified as Black or African American; 708 trainees (5.1%) identified as Hispanic or Latino; 22 trainees (0.2%) identified as Native American, American Indian or Alaskan native; 7219 trainees (52.1%) identified as White; and 1069 trainees (7.7%) identified as other. Only 1250 (9.0%) of 13 853 trainees were URM individuals.

URM representation in oncology was significantly lower than all other specialties across all study years combined (9.0% [95% CI, 8.5%-9.5%] vs 13.1% [95% CI, 13.1%-13.2%]; *P* = .001). Of the oncology specialty groups, only gynecologic oncology (22.7% [95% CI, 8.2%-39.1%]; *P* = .02) and hematology and medical oncology (3.0% [95% CI, 0.4%-5.7%]; *P* = .04] demonstrated significant positive AAPCs (Table and Figure).

**Table.** Absolute Percentage Change in URM Representation and AAPC Across Study Years

Specialty	URM, % (95% CI)		Absolute % change	AAPC (95% CI)	*P* value^a^
	First year	Last year			
All oncology specialties	8.9 (7.8 to 10.0)	9.7 (8.7 to 10.8)	+0.8	+1.9 (−2.9 to 7.0)	.30
Hematology and oncology	8.4 (6.9 to 9.9)	9.6 (8.2 to 11.0)	+1.2	+3.0 (0.4 to 5.7)	.04^a^
Gynecologic oncology	7.9 (3.8 to 12.0)	13.9 (9.1 to 18.7)	+6.0	+22.7 (8.2 to 39.1)	.02^a^
Pediatric hematology and oncology	12.3 (9.1 to 15.4)	12.6 (9.5 to 15.7)	+0.3	−0.3 (−13.5 to 14.9)	.95
Radiation oncology	7.6 (5.6 to 9.7)	7.3 (5.4 to 9.3)	−0.3	−1.3 (−4.2 to 1.8)	.28
Complex general surgical oncology	10.1 (3.8 to 16.4)	6.1 (1.4 to 10.9)	−4.0	−8.7 (−20.2 to 4.5)	.12
All other specialties	13.0 (12.7 to 13.2)	13.5 (13.3 to 13.7)	+0.5	+1.1 (0.2 to 1.9)	.03^a^

Abbreviations: AAPC, average annual percentage change; URM, underrepresented minority (ie, Black or African American individuals, Hispanic or Latino individuals, and Native American, American Indian, or Alaskan Native individuals).

^a^ Represents significance at *P* < .05.

**Figure.**
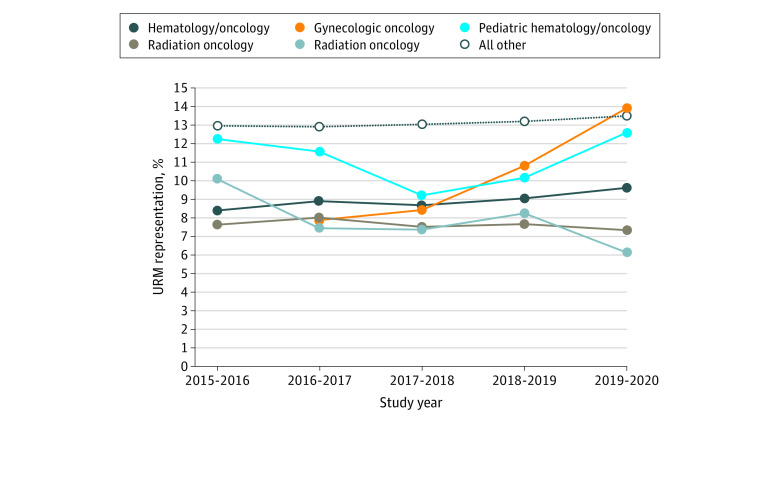
Representation Trends Across Oncology Specialty Groups From 2015-2016 to 2019-2020

## Discussion

To our knowledge, this study is the first to investigate URM representation trends among oncology training programs in the US. It found that the continued disparities in URM representation exist within oncology training.^3^ From 2015 to 2020, only 3.8% of oncology trainees identified as Black or African American, 5.1% as Hispanic or Latino, and 0.2% as Native American, American Indian, or Alaskan Native, while individuals identifying as these races and ethnicities make up 13%, 18%, and 1.3% of the total US population in the 2020 census, respectively. This finding is consistent with studies demonstrating persistent URM underrepresentation across both surgical and nonsurgical specialties.^2^

One limitation of this study was the exclusion of residents of unknown racial/ethnic identity, because URM individuals are more likely to report as unknown. Trainees are also counted in multiple years because of the nature of residency programs. Furthermore, because of ACGME changes, this study does not include Pacific Islander and Native Hawaiian groups in the definition of URM, and individuals who identified as Spanish origin were included in the Hispanic or Latino groups in 2019 to 2020.

It is important to consider these results in the context of persistently low rates of URM representation in graduate medical education because of structural racism and other potential factors, which limit representation in training programs across all specialties.^2,6^ However, rates of representation in oncology remain significantly lower than all other specialties combined. Potential contributing factors to this disparity, including trainee preferences for specialties in primary care, length of training, recruitment strategies, pipeline specialty demographics, and specialty workforce demographics, must be studied further in order to address this disparity and create a diverse and representative workforce that meets the health needs of the diverse populations served.
