# Error in Figure Key

**DOI:** 10.1001/jamanetworkopen.2021.37690

**Published:** 2021-11-05

In the Research Letter titled “Trends in Race and Ethnicity Among Matriculants to US Oncology Training Programs, 2015-2020,”^1^ published October 7, 2021, there was an error in the Figure key. Two of the lines in the Figure were labeled radiation oncology while 1 of the lines should have been labeled complex general surgery oncology. This article has been corrected.^1^
